# Automated assessment of CD8^+^ T-lymphocytes and stroma fractions complement conventional staging of colorectal cancer

**DOI:** 10.1016/j.ebiom.2021.103547

**Published:** 2021-08-31

**Authors:** Dan Jiang, Tarjei S. Hveem, Mark Glaire, David N. Church, David J. Kerr, Li Yang, Håvard E. Danielsen

**Affiliations:** a Sichuan University-University of Oxford Huaxi Joint Centre for Gastrointestinal Cancer, West China Hospital, Sichuan University, Chengdu, China; b Department of Pathology, West China Hospital, Sichuan University, Chengdu, China; c Radcliffe Department of Medicine, University of Oxford, Oxford, United Kingdom; d Institute for Cancer Genetics and Informatics, Oslo University Hospital, Oslo, Norway; e Wellcome Centre for Human Genetics, University of Oxford, Oxford, United Kingdom; f NIHR Oxford Biomedical Research Centre, Oxford University Hospitals NHS Foundation Trust, John Radcliffe Hospital, Oxford, United Kingdom; g Department of Informatics, University of Oslo, Oslo, Norway; h Department of Gastroenterology and Hepatology, West China Hospital, Sichuan University, Chengdu, Sichuan, China

**Keywords:** Colorectal cancer, Prognosis, CD8, Stroma

## Abstract

**Background:**

Tumor development is critically dependent on the supporting stroma consisting of inflammatory cells and fibroblasts. This study intended to improve prognostic prediction for early colorectal cancer (CRC) by combined estimation of T-lymphocyte and stroma fractions with conventional markers.

**Methods:**

In total 509 and 1041 stage II/ΙΙΙ CRC from the VICTOR and QUASAR 2 trials were included as a training set and a validation set, respectively. Intratumoral CD8^+^ T-lymphocytes and stroma were identified and quantified by machine-based learning on digital sections. The primary endpoint was to evaluate the prognostic value of the combined marker for time to recurrence (TTR).

**Findings:**

For low-risk patients (*n* = 598; stage Ⅱ, and stage ΙΙΙ pT1-3 pN1 with neither lymphatic (L^−^) nor vascular (V^−^) invasion), low stroma fraction (*n* = 511) identified a good prognostic subgroup with 5-year TTR of 86% (95% CI 83–89), versus the high stroma subgroup TTR of 78% (HR = 1.75, 95% CI 1.05–2.92; *P* = 0.029). For high-risk patients (*n* = 394; stage ΙΙΙ pT3 pN1 L^+^/V^+^, pT4, or pN2), combined low CD8^+^ and high stroma fraction identified a poor prognostic subgroup (*n* = 34) with 5-year TTR of 29% (95% CI 17-50), versus the high CD8^+^ fraction and low stroma fraction subgroup (*n* = 138) of 64% (HR = 2.86, 95% CI 1.75–4.69; *P* < 0.001).

**Interpretation:**

Quantification of intratumoral CD8^+^ T-lymphocyte and stroma fractions can be combined with conventional prognostic markers to improve patient stratification.

**Research in context**

**Evidence before this study**

There is a significant concern about refining prognostic predication to provide more precise treatment strategies for early-stage colorectal cancer (CRC). We searched Pubmed using the search terms of “colorectal cancer”, “stroma”, “CD8”, and “prognosis”. A positive correlation between CD8+ T-lymphocyte and prognosis has been reported in many studies. The tumours carrying more than 50% intratumoural stroma had a poor prognosis. An immune scoring system has been proposed in a previous study to predict the prognosis of CRC, which was based on the quantitative total CD3+ T-lymphocytes and cytotoxic CD8+ T-lymphocytes density. No study has yet tested the prognostic prediction power of a combination of markers that integrate tumour-infiltrating immune cells, tumour stroma, and conventional prognostic markers in large well-curated trials. Therefore we applied two mature clinical trials, VICTOR and QUASAR 2 to address this question.

**Added value of this study**

Low stroma fraction combined with low-risk clinicopathological markers can identify a good prognostic subgroup with 5-year time to recurrence (TTR) of 86%. Low CD8+ T-lymphocyte fraction and high stroma fraction combined with high-risk clinicopathological parameters can identify a very poor prognostic subgroup CRC with 5-year TTR of only 29% and 5-year overall survival of 42%.

**Implications of all the available evidence**

Our data can objectively identify specific prognostic subgroups of early-stage CRC using a machine learning algorithm applied to digital images and may provide evidence to integrate this prognostic marker into the conventional Tumour, Node and Metastases (TNM) stage report.

Alt-text: Unlabelled box

## 1 Introduction

Colorectal cancer (CRC) is one of the most common cancer diagnoses and is a leading cause of cancer-related deaths worldwide [1]. The prognostic prediction of patients with CRC relies on American Joint Committee on Cancer (AJCC) and Union for International Cancer Control (UICC) Tumor, Node and Metastases (TNM) staging system [2]. However, the patients within the same broad Tumor (T) & Node (N) stage often have a range of different clinical outcomes [3]. For stage Ⅱ/ΙΙΙ CRC, which accounts for more than half of CRC at initial diagnosis, further risk evaluation is usually based on DNA mismatch repair/microsatellite instability (MMR/MSI) status combined with several pathological and clinical risk factors (e.g. lymphatic/vascular invasion, bowel obstruction, and <12 lymph nodes harvested), but these markers are considered individually rather than as an integrated whole, influenced both by how these data are presented in structured pathology reports and by a tendency in the literature to present any novel biomarker as a ‘stand alone’ indicator [1,3,4].

Emerging evidence indicates that the tumor microenvironment (TME) plays a crucial role in tumor progression in CRC with several cell types influencing each other's phenotype through direct intercellular communication and the elaboration of multiple chemokines and cytokines [5]. This specialized stroma is characterized by increased production of extracellular matrix proteins and paracrine growth factors, endothelial cells, inflammatory cells (lymphocytes, tumor associated macrophages and neutrophils) and cancer associated-fibroblasts [5]. This represents a biologically, complex interactive system in which it is extremely difficult to apportion the contribution of individual cell types or cytokines to the overall tumor phenotype. Given the resurgence of interest in immunotherapy, there has been an increasing focus on tumor-infiltrating T-lymphocyte density and assessment of the total stroma/epithelial cell ratio has also been identified as an independent prognostic biomarker in CRC [[6], [7], [8]]. Although lymphocytes comprise an important subset of the stroma, it was hypothesized in the present study that a combination of these two markers will further refine the prognosis.

In the present study, we analyzed the prognostic ability of the novel combination of CD8^+^ T-lymphocyte fraction and stroma fraction using digital pathology based automated assessment and integrated these parameters with conventionally accepted prognostic factors (lymphovascular invasion, *T* and *N* staging) in a large discovery dataset, which was subsequently validated by multivariate modeling in a larger cohort of stage Ⅱ/ΙΙΙ CRC patients in the QUASAR 2 clinical trial [9].

## 2 Methods

### 2.1 Patients and treatment

In total 509 stage Ⅱ and stage ΙΙΙ CRC patients from VICTOR trial (a phase 3 clinical trial, registered number NCT00031863) were included in the training set where optimal CD8^+^ and stroma thresholds were identified [10]. The inclusion of the 509 cases were based on the availability of digital slides of tumor tissue and clinical outcome data. In the VICTOR trial, a total of 2434 patients with stage Ⅱ or ΙΙΙ CRC were randomly assigned to receive rofecoxib (a cyclooxygenase-2 inhibitor) or placebo. There was no difference in recurrence rate and overall survival (OS) between the two groups.

QUASAR 2, a large phase 3 clinical trial, whose details have been reported previously (registered number ISRCTN45133151) was used as a validation set [9]. Briefly, 1941 histologically proven high-risk stage Ⅱ (i.e. with one or more of the following features: stage T4, lymphatic or vascular invasion, peritoneal involvement, poor differentiation and obstruction or perforation of the primary tumor) or stage ΙΙΙ patients with surgically resected CRC were randomly assigned to be treated with capecitabine (CAP) alone or capecitabine plus bevacizumab (CAP+BEV) after pathologically confirmed R0 resection. Disease-free and overall survival at 3 years did not significantly differ between the two treatment groups. In this present study, 1041 cases were included from the QUASAR 2 trial based on the availability of tumor tissue and clinical outcome data.

### 2.2 CD8 immunohistochemistry (IHC) and evaluation

Tissue microarray (TMA), tissue slides, CD8 IHC and cell number quantification were performed as previously described [11]. After exclusion of TMA cores with less than 1000 cells, IHC of CD8 (Leica Biosystems Cat# NCL-CD8-4B11, RRID:AB_442068) was performed on duplicated or triplicated TMA tumor cores.

The fraction of CD8^+^ T-lymphocyte was calculated as the number of CD8 positive cells in each TMA core compared to the total cells number. A final mean_fraction was used, which was the average of the fractions of the duplicated or replicated TMA cores for each case. Quantification of the CD8 positive cell number and the total number of cells was performed by computerized digital image analyses using ImmunoPath1.4.15.0 (Room4, Crowborough, UK) as previously described [11,12].

The development of software was described in the previous study [12]. Briefly, Circular Hough Transform is used to detect the nuclei, which are classified as positive (brown) or negative (blue) based on HSV (Hue, Saturation and Value) thresholds. Images from nine of the cases (representing a diversity of the material with respect to staining intensity and fraction) were used to adjust HSV based threshold manually to get the correct color ranges for both positive and negative nuclei. The color ranges were refined until visually acceptable results were achieved and an image analysis protocol was set up and run for all TMA cores. Each slide was scanned by the Hamamatsu Digital Slide Scanner NanoZoomer 2.0-HT C9600-13.

**Fig. 1:**
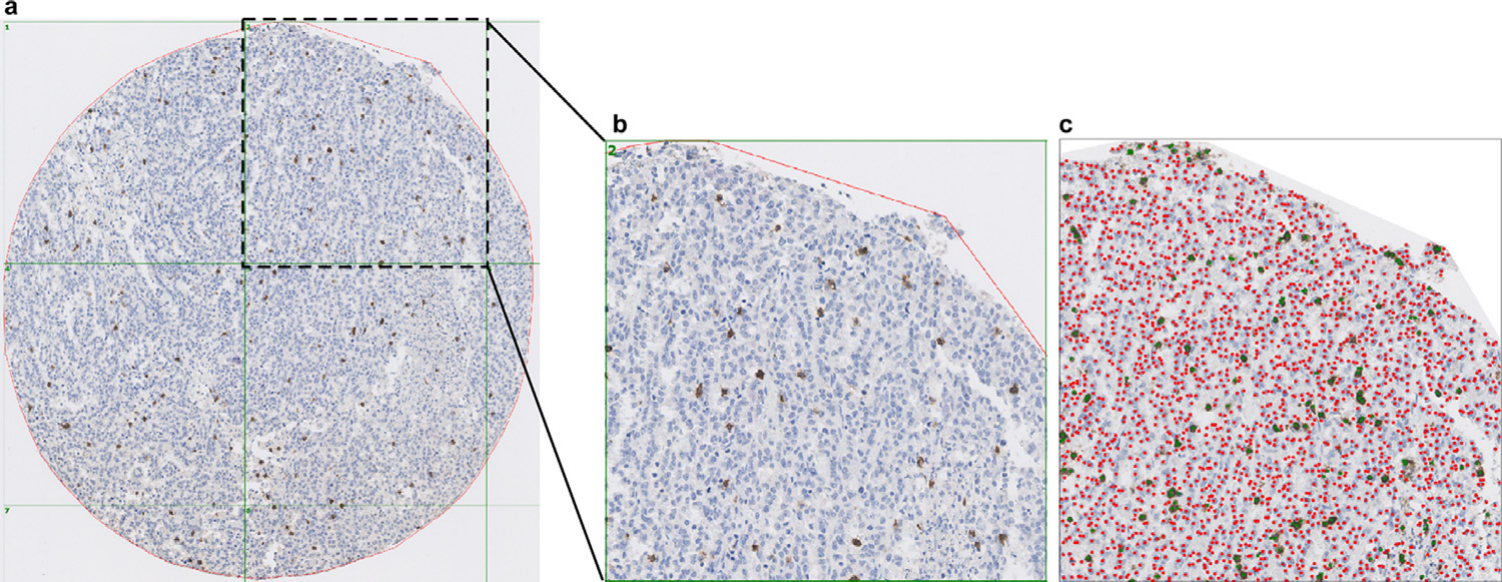
**Computerized image analysis of CD8^+^ T-lymphocytes in digital tissue slides.** (a) Overview of one representative tissue core with CD8 (Leica Biosystems Cat# NCL-CD8-4B11, RRID:AB_442068) immunohistochemical staining. (b) Positive (brown) and negative (counterstained blue) nuclei were shown on magnified image of one subarea. (c) Computerized image analysis was performed by using the software (Immunopath), where positive nuclei were marked with green dots, whereas the negative nuclei were marker with red dots. (For interpretation of the references to color in this figure legend, the reader is referred to the web version of this article).

Representative CD8 IHC staining images are presented in Fig. 1.

### 2.3 Stroma identification and fraction analysis

Haematoxylin and eosin (H&E) stained whole tissue slides were scanned at x40 with an Aperio AT2 digital slide scanner (Leica Biosystems, Germany). Tumor area was annotated by a pathologist for each slide. The stroma fraction was estimated within the annotated regions of a scan, where each scan was analyzed separately as described below using a software tool (Stroma Analyzer, Room4 Group Ltd, Crowborough, UK) [13].

The image of all pixels within the annotated regions of a scan was corrected for white balance using Huo's method, an iterative method operating in YUV color space using automatically extracted grey color points [14]. Subsequently, the RGB (Red-Green-Blue) intensities (range 0–255) were scaled by multiplication with the factor required to set the 95% percentile in the grey level intensity converted image to 240. Two stain vectors that were representative of the saturation of the H&E stains in the corrected image were automatically identified and utilized to normalize and perform color deconvolution using a previously described method [15]. Subsequently, two normalized images were extracted; one of the haematoxylin component (normalized haematoxylin image) and one of the H&E components combined (normalized H&E image). Two masks were then created for the automatic detection of background, stroma and epithelial tissue.

To compute a background mask that identifies regions without tissue, an averaging filter of size 7 × 7 pixels was applied to the normalized H&E image in each of the RGB color channels individually. The filtered image was then converted to the HSV color space and foreground pixels were defined as pixels with a value (V channel) greater than or equal to 0.2 and a saturation (S channel) greater than or equal to 0.4.

To compute a stroma mask that distinguish stroma tissue from epithelial tissue, the normalized haematoxylin image was converted from the RGB color space to greyscale using Rx0.3 + Gx0.6 + Bx0.1 and median filtered with a filter mask of 9 × 9 pixels. A sample standard deviation filter with 17 × 17 pixels was applied to the median filtered image. The median filtered image and the standard deviation filtered image were shifted and scaled such that resulting intensity values spanned the full intensity range (i.e. 0 to 255) in each image, where values lower than the 1% percentile and higher than the 99% percentile were set to 0 and 255, respectively. The resulting images were combined into one image by averaging the values for each pixel. Otsu's method, which estimates the optimum threshold between two classes as the threshold level where the variance within the two classes was at its minimum, was used to calculate the threshold used to distinguish stroma from epithelial tumor [16]. The calculated value was scaled by 1.15 and used to threshold the combined image. Connected regions with <200 foreground pixels were removed and morphological closing with a structure element of 5 × 5 pixels was performed.

The stroma mask and the background mask were then combined by a logical AND operation. The area of stroma and epithelial tissue within the annotated area was estimated using this final mask and stroma fraction was calculated by dividing the stroma area (in pixels) by the combined area of stroma and epithelial tissue.

Calibration factors cf1 and cf2 specific to scanner types were calculated using linear regression from a set of scans scanned on both scanners (Aperio AT2 scanner cf1 and cf2). Factors were applied to obtain a scanner invariant result, corrected stroma fraction: (stroma_fraction-cf1)/cf2. For the Aperio AT2 scanner, cf1 and cf2 were 0.037 and 0.9852, respectively.

**Fig. 2:**
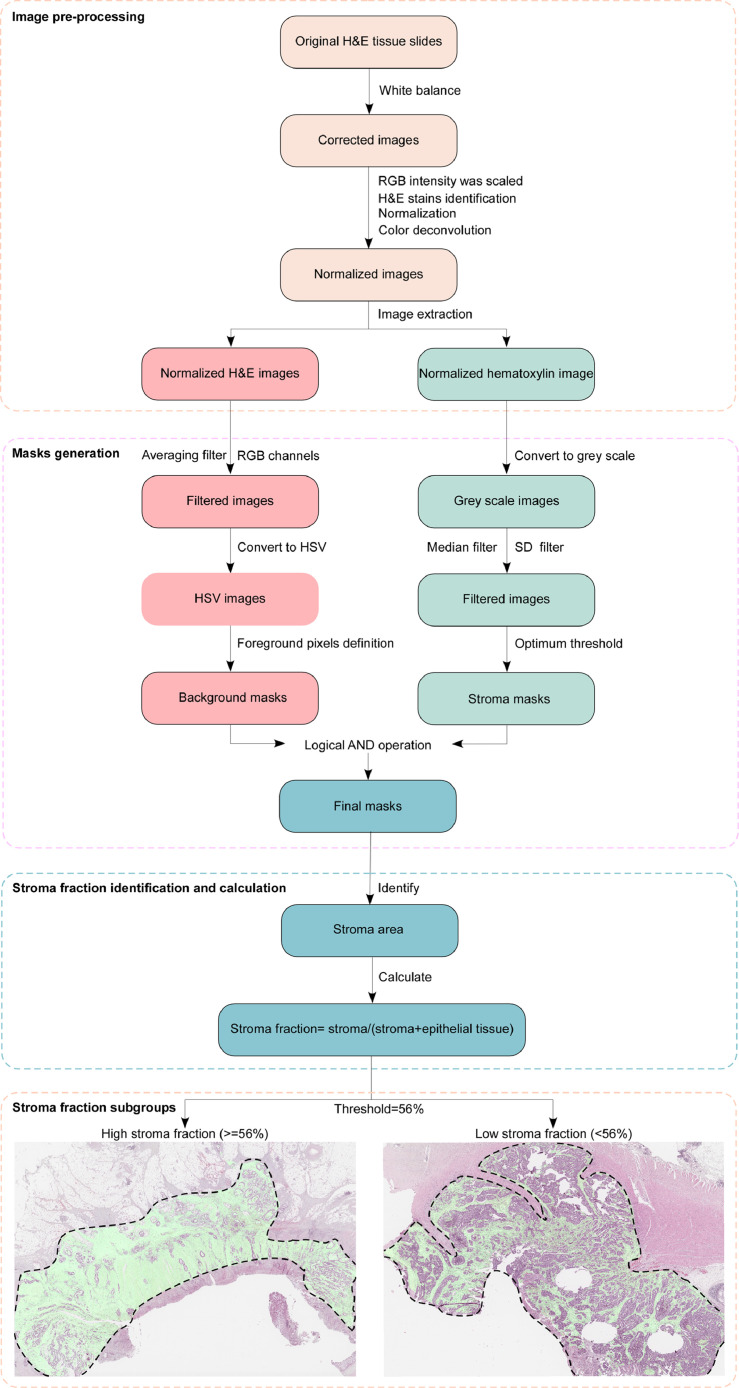
**Schematic diagram of stroma analysis process.** In the haematoxylin and eosin (H&E) stained whole tissue slide images, the dotted line indicates the tumor area, and within the tumor area, the green area is the stromal area identified by the machine-based analysis. Abbreviations: RGB, Red-Green-Blue; HSV, Hue-Saturation-Value; SD, standard deviation. (For interpretation of the references to color in this figure legend, the reader is referred to the web version of this article).

A schematic diagram is shown in Fig. 2.

### 2.4 Identification thresholds of CD8^+^ and stroma fraction

In order to identify the optimal combination of CD8^+^ and stroma fraction thresholds, the lower and upper thresholds for each of the two parameters were first identified that when used to dichotomize the patient group provided at least five patients in the smallest group. The CD8^+^ fractions were evaluated in steps of 0.001 whereas the stroma fractions were evaluated in steps of 0.01. For CD8^+^ the lower threshold satisfying this constraint was 0.001 and the upper was 0.158; consequently, the CD8^+^ thresholds evaluated were 0.001 to 0.158. For stroma the lower threshold was 0.26 and the upper was 0.71; consequently, the stroma thresholds evaluated were 0.26 to 0.71.

A patient was classified into one of three groups based on the CD8^+^ and stroma thresholds:

- High CD8^+^ and low stroma when the CD8^+^ value was higher than the CD8^+^ threshold and the stroma value was lower than the stroma threshold.
- Low CD8^+^ or high stroma when the CD8^+^ value was lower than or equal to the CD8^+^ threshold or the stroma value was higher than or equal to the stroma threshold.
- Low CD8^+^ and high stroma when the CD8^+^ value was lower than or equal to the CD8^+^ threshold and the stroma value was higher than or equal to the stroma threshold.

There were a total of 7268 threshold combinations (158 CD8^+^ thresholds multiply by 46 stroma thresholds equal 7268 threshold combinations). For each threshold combination, we evaluated the CD8^+^ and stroma marker categorised in three levels as described above in univariable Cox regression and calculated the concordance index (*C*-index). The combination with the highest C-index was selected for validation. This combination was a CD8^+^ threshold of 0.027 and a stroma threshold of 0.56.

**Fig. 3:**
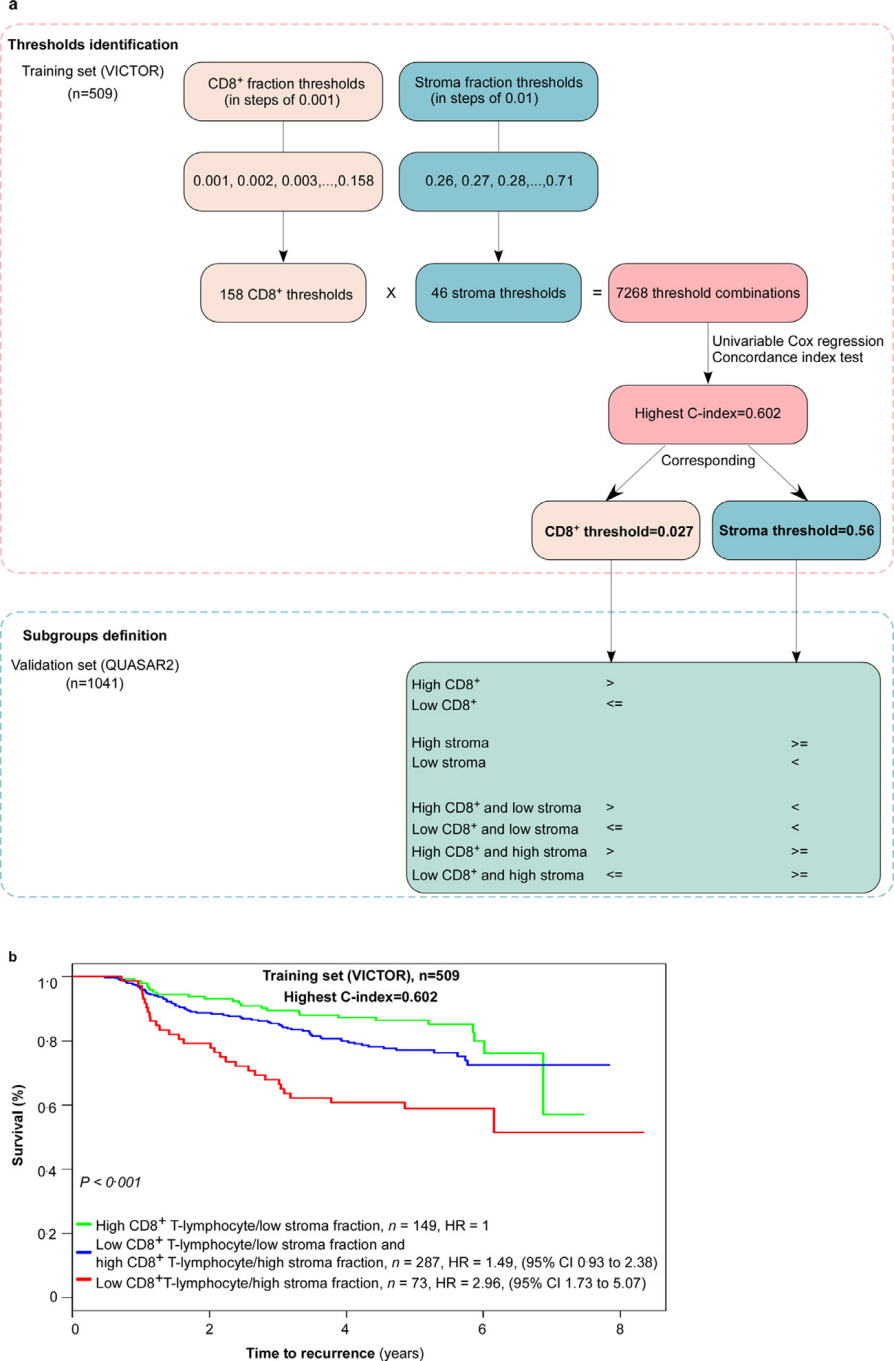
**The process of CD8^+^ T-lymphocytes and stroma fraction thresholds identification and subgroups definition.** (a) Schematic diagram of CD8^+^ T-lymphocytes and stroma fraction thresholds identification and subgroups definition. (b) Kaplan-Meier plot where the CD8^+^ and stroma thresholds for the highest concordance index (*C*-index) are used to categorize patients in the training set.

Fig. 3 shows thresholds identification and subsequent subgroups classification, and the 5-year TTR survival curves for the training set at the highest *C*-index.

### 2.5 Microsatellite instability (MSI), KRAS and BRAF analysis

MSI was analyzed utilizing five Bethesda microsatellite markers (BAT25, BAT26, D2S123, D5346, and D17S250) plus BAT40. Tumors with 40% or more unstable markers were classified as being microsatellite instability-high (MSI-H), and otherwise as microsatellite stable (MSS).

Standard direct DNA sequencing was performed for KRAS (exon 2). Further sequencing for KRAS exon 3 was done in 458 tumors, although <1% were mutant. For BRAF, mutations were initially screened in 464 tumors by direct DNA sequencing, and after that using a KASPar genotyping assay designed for the hotspot T1799A (V600E), which had shown excellent sensitivity and specificity in our previously-sequenced samples [17].

### 2.6 Statistical analysis

The primary endpoint was time to recurrence (TTR), defined as time from randomization to the date of local or regional recurrence, distant recurrence, or to the date of death from CRC (second primary same cancers and other primary cancers were ignored. Deaths from other cancers, non-cancer-related deaths, treatment-related deaths, and loss to follow-up were censored observations). The secondary outcome of interest was OS, calculated from the time of randomization to death from any cause or the last date of follow-up. Survival distributions were compared using the Mantel-Cox log-rank test in univariable analyses of categorical variables and the Wald's chi-squared test in univariable analyses of continuous variables and in multivariable analyses. Kaplan-Meier curves were used to illustrate survival probability for patients grouped by the proposed marker. Patients with missing values for any included variable were excluded from the multivariable analyses. Analyses were performed using *R*, Version3.5.2. Two-sided *P* < 0.05 was considered statistically significant.

### 2.7 Ethics

All participants provided written informed consent for use of tumor tissue and blood samples, and the study was approved by medical ethics committees at all sites.

### 2.8 Role of the funding source

The funders of the study had no role in study design, data collection, data analysis, data interpretation or writing the report. All authors had full access to the data and the corresponding authors had the final responsibility to submit for publication.

## 3 Results

### 3.1 Patient demographics and clinical characteristics

The median follow-up time for the training set (*n* = 509) was 5.1 (interquartile range 4–5.8) years. 241 patients (47%) patients had stage II CRC, and 268 (53%) patients had stage III CRC. 119 (23%) patients had a relapse, and 90 (18%) patients died.

The median follow-up time for the validation set (*n* = 1041) was 4.7 (interquartile range 3.4–5.1) years, 369 (35%) patients had stage II CRC, and 672 (65%) patients had stage III CRC. 256 (25%) patients had a relapse, and 205 (20%) patients died. The two cohorts were well balanced in demographic and clinicopathological characteristics. The distribution of clinicopathological variables in the training set and validation set was summarized in Table 1.

**Table 1:** Clinicopathological data of the training set and the validation set.

**Variable**	**Training set (*n =* 509)**	**Validation set (*n =* 1041)**
	**n(%)**	
Age (years)	Median 65 (IQR 58–64)	Median 65 (IQR59-71)
Gender		
Female	181 (36%)	437 (42%)
Male	328 (64%)	604 (58%)
Stage		
II	241 (47%)	369 (35%)
III	268 (53%)	672 (65%)
pT stage		
pT1	7 (1%)	14 (1%*)*
pT2	38 (7%)	65 (6%)
pT3	352 (69%)	542 (52%)
pT4	100 (20%)	374 (36%)
Missing	12 (2%)	46 (4%)
pN stage		
pN0	236 (46%)	356 (34%)
pN1	186 (37%)	477 (46%)
pN2	74 (15%)	167 (16%)
Missing	13 (3%)	41 (4%)
Lymphatic invasion		
No	444 (87%)	885 (85%)
Yes	48 (9%)	96 (9%*)*
Missing	17 (3%)	60 (6%)
Venous vascular invasion		
No	395 (78%)	589 (57%)
Yes	97 (19%)	400 (38%)
Missing	17 (3%)	52 (5%)
Sidedness		
Right	205 (40%)	569 (55%)
Left	291 (57%)	424 (41%)
Missing	13 (3%)	48 (5%)
Microsatellite instability		
MSI-H	63 (12%)	124 (12%)
MSS	436 (86%)	874 (84%)
Missing	10 (2%)	43 (4%)
KRAS		
Wild type	334 (66%)	643 (62%)
Mutated	171 (34%)	314 (30%)
Missing	4 (1%)	84 (8%)
BRAF		
Wild type	454 (89%)	836 (80%)
Mutated	54 (11%)	127 (12%)
Missing	1 (0%)	78 (8%)
CD8^+^ T-lymphocyte fraction		
Low (<=2.7%)	208 (41%)	511 (51%)
High (>2.7%)	301 (59%)	530 (49%)
Stroma fraction		
Low (<56%)	377 (74%)	882 (85%)
High (>=56%)	132 (26%)	159 (15%)

Abbreviations: IQR, interquartile range; pT stage, pathological tumor stage; pN stage, pathological lymph node stage; MSI-H, microsatellite instability-high; MSS, microsatellite stable.

High and low risk categories were aggregated after multivariate modeling (Table 2) of conventionally accepted prognostic markers into the following groups: stage III high risk patients - pT3 with either lymphatic (L^+^) and/or vascular (V^+^) invasion, pT4 or pN2; stage III low risk patients - pT1-3 pN1 L^−^ V^−^.

**Table 2:** Univariate and multivariable analyses of 5-year time to recurrence according to clinicopathological features in the validation set of patients.

			**Stage II**						**Stage III**			
			**Univariable analyses**		**Multivariable analyses**				**Univariable analyses**		**Multivariable analyses**	
**Variable**	**Total number**	***n* (%)**	**HR (95% CI)**	***P*-value**	**HR (95% CI)**	***P*-value**	**Total number**	***n* (%)**	**HR (95% CI)**	***P*-value**	**HR (95% CI)**	***P-*value**
Age	369	369 (100%)	1.00 (0.98–1.03)	0.78	1.00 (0.97–1.03)	0.97	672	672 (100%)	1.00 (0.99–1.01)	0.98	0.99 (0.98–1.01)	0.39
Gender	369			0.61			672			0.45		
Male		217 (59%)	Ref.					387 (58%)	Ref.			
Female		152 (41%)	0.87 (0.51–1.49)					285 (42%)	0.90 (0.68–1.19)			
Randomized adjuvant treatment	369			0.041		0.015	672			0.12		
CAP		181 (49%*)*	Ref.		Ref.			330 (49%)	Ref.			
CAP+BEV		188 (51%)	1.75 (1.02–3.00)		2.08 (1.15–3.73)			342 (51%)	1.25 (0.95–1.65)			
Sidedness	353			0.26			640			0.12		
Right		175 (50%)	Ref.					394 (62%)	Ref.			
Left		178 (50%)	0.73 (0.42–1.27)					246 (38%)	1.25 (0.94–1.67)			
pT stage	354			0.062			641			<0.001		<0.001
pT1								14 (2%)	Ref.		Ref.	
pT2								65 (10%)	1.32 (0.30–5.92)		1.52 (0.34–6.83)	
pT3		177 (50%)	Ref.					365 (57%)	1.72 (0.42-6.98)		1.29 (0.32-5.30)	
pT4		177 (50%)	1.69 (0.97–2.96)					197 (31%)	4.87 (1.20–19.74)		3.16 (0.77–12.95)	
Number of lymph nodes removed	354			0.018		0.015	642			0.21		
>=12		289 (82%)	Ref.		Ref.			481 (75%)	Ref.			
<12		65 (18%)	2.05 (1.12–3.75)		2.12 (1.16–3.89)			161 (25%)	1.22 (0.89–1.66)			
pN stage							644			<0.001		<0.001
pN1								477 (74%)	Ref.		Ref.	
pN2								167 (26%)	3.35 (2.53–4.44)		2.40 (1.77–3.24)	
Histological grade	345			0.47			635			0.14		
Well		16 (5%)	Ref.					26 (4%)	Ref.			
Moderate		264 (77%)	0.84 (0.26–2.70)					517 (81%)	2.05 (0.76–5.54)			
Poor		65 (19%)	0.51 (0.13–2.02)					92 (14%)	2.62 (0.93–7.41)			
Venous vascular invasion	350			0.81			639			<0.001		0.011
No		197 (56%)	Ref.					392 (61%)	Ref.		Ref.	
Yes		153 (44%)	0.93 (0.54–1.62)					247 (39%)	2.10 (1.58–2.79)		1.48 (1.10–1.99)	
Lymphatic invasion	348			0.47			633			<0.001		0.002
No		317 (91%)	Ref.					568 (90%)	Ref.		Ref.	
Yes		31 (9%)	1.37 (0.58–3.21)					65 (10%)	2.25 (1.55–3.26)		1.80 (1.24–2.63)	
KRAS	342			0.17			615			0.89		
Wild type		240 (70%)	Ref.					403 (66%)	Ref.			
Mutated		102 (30%)	1.47 (0.84–2.57)					212 (34%)	1.02 (0.75–1.39)			
BRAF	338			0.64			625			0.11		
Wild type		291 (86%)	Ref.					545 (87%)	Ref.			
Mutated		47 (14%)	0.82 (0.35–1.91)					80 (13%)	1.39 (0.93–2.08)			
Microsatellite instability	353			0.14			645			0.10		
MSI-H		58 (16%)	Ref.					66 (10%)	Ref.			
MSS		295 (84%)	1.96 (0.78–4.93)					579 (90%)	1.59 (0.91–2.80)			
CD8^+^ T-lymphocyte and stroma fraction	369			0.15			672			<0.001		0.001
High CD8^+^ fraction and low stroma fraction		186 (50%)	Ref.					259 (39%)	Ref.		Ref.	
Low CD8^+^ fraction or high stroma fraction		158 (43%)	1.57 (0.90–2.74)					364 (54%)	1.34 (0.98–1.83)		1.30 (0.94–1.79)	
Low CD8^+^ fraction and high stroma fraction		25 (7%)	2.09 (0.85–5.16)					49 (7%)	2.93 (1.86–4.60)		2.50 (1.57–3.97)	
CD8^+^ T-lymphocyte fraction	369			0.26			672			0.032		
High (>2.7%)		222 (60%)	Ref.					308 (46%)	Ref.			
Low (<=2.7%)		147 (40%)	1.35 (0.80–2.28)					364 (54%)	1.36 (1.03–1.81)			
Stroma fraction	369			0.072			672			<0.001		
Low (<56%)		308 (83%)	Ref.					574 (85%)	Ref.			
High (>=56%)		61 (17%)	1.73 (0.94–3.17)					98 (15%)	1.92 (1.38–2.69)			

Abbreviations: HR, hazard risk; CI, confidence interval; Ref, reference; CAP, capecitabine; BEV, bevacizumab; pT stage, pathological tumor stage; pN stage, pathological lymph node stage; MSI-H, microsatellite instability-high; MSS, microsatellite stable.

### 3.2 MSI, KRAS, and BRAF status

In the validation set, 12% tumors were MSI-H, 30% carried KRAS mutation and 12% BRAF mutations. MSI status, mutant KRAS and BRAF were not significant predictors of recurrence in neither stage Ⅱ nor stage III CRC (Table 2).

### 3.3 Stage Ⅱ and low-risk stage III CRC (pT1-3 N1 L^−^ V^−^)

**Fig. 4:**
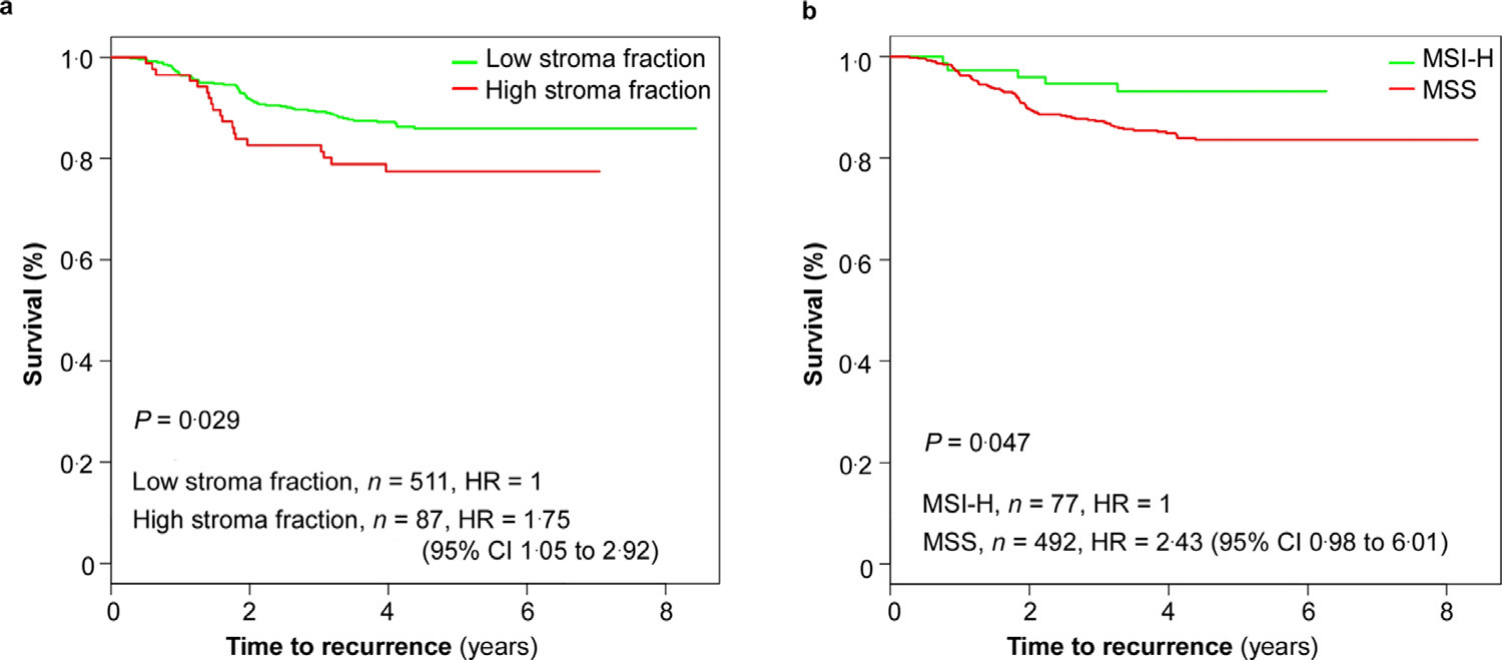
**Kaplan-Meier curves for time to recurrence (TTR) according to stroma fraction or microsatellite instability (MSI) status in the low-risk subgroup of the validation set.** (a) Kaplan-Meier curves for TTR according to the stroma fraction in stage Ⅱ and low-risk stage ΙΙΙ colorectal cancer (CRC), *n =* 598. 5-year TTR: 86% (95% CI 83–89) for patients with low stroma fraction versus 78% (95% CI 69–87) for patients with high stroma fraction (Mantel-Cox log-rank test, *P* = 0.029). (b) Kaplan-Meier curves for TTR according to MSI status in stage Ⅱ and low-risk stage ΙΙΙ CRC, *n =* 569. 5-year TTR: 93% (95% CI 87–99) for patients with MSI-H (microsatellite instability-high) versus 84% (95% CI 80–87) for patients with MSS (microsatellite stable) (Mantel-Cox log-rank test, *P* = 0.047). Low-risk stage ΙΙΙ CRC: pathological tumor stage (pT) 1–3 pathological lymph node stage (pN) 1 without lymphovascular invasion (L^−^) nor venous vascular invasion (V^−^). HR, hazard risk; CI, confidence interval.

In early stage CRC (*n* = 598, all stage II, and stage III pT1-3N1 L^−^ V^−^), which collectively defines a relatively good prognosis with 5-year OS of around 79% (95% CI 76-82), the stroma fraction provided useful separation in risk of recurrence and survival. Low stroma fraction (*n* = 511) identify a subgroup with 5-year TTR of 86% (95% CI 83-89), compared to 78% (95% CI 69-87) in the group with high stroma fraction (*n* = 87, HR = 1.75, 95% CI 1.05–2.92; Mantel-Cox log-rank test, *P* = 0.029; Fig. 4, Table 3). MSI-H identified a subgroup with low-risk tumor recurrence with 5-year TTR of 93% (95% CI 87-99; Fig. 4, Table 3). None of these markers were useful for 5-year OS prediction (Supplementary Table 1).

**Table 3:** Univariate analyses of 5-year time to recurrence according to clinicopathological features in in the validation set of patients.

		Stage ΙΙ and low-risk stage ΙΙΙ				High-risk stage ΙΙΙ		
Variable	Total number	*n* (%)	HR (95% CI)	*P*-value	Total number	*n* (%)	HR (95% CI)	*P*-value
Age	598	598 (100%)	1.00 (0.98–1.03)	0.80	394	394 (100%)	1.00 (0.98–1.01)	0.61
Gender	598			0.49	394			0.88
Male		342 (57%)	Ref.			234 (59%)	Ref.	
Female		256 (43%)	0.86 (0.55–1.32)			160 (41%)	1.02 (0.75–1.40)	
Randomized adjuvant treatment	598			0.016	394			0.17
CAP		295 (49%)	Ref.			197 (50%)	Ref.	
CAP+BEV		303 (51%)	1.71 (1.10–2.65)			197 (50%)	1.24 (0.91–1.69)	
Sidedness	578			0.11	387			0.081
Right		321 (56%)	Ref.			226 (58%)	Ref.	
Left		257 (44%)	0.69 (0.44–1.09)			161 (42%)	1.32 (0.97–1.81)	
Number of lymph nodes removed	583			<0.001	393			0.39
>=12		453 (78%)	Ref.			307 (78%)	Ref.	
<12		130 (22%)	2.28 (1.45–3.60)			86 (22%)	1.17 (0.82–1.66)	
Histological grade	572			0.84	383			0.43
Well		25 (4%)	Ref.			13 (3%)	Ref.	
Moderate		461 (81%)	0.85 (0.31–2.33)			302 (79%)	2.08 (0.66–6.52)	
Poor		86 (15%)	0.72 (0.23–2.31)			68 (18%)	2.16 (0.66–7.10)	
KRAS	555			0.18	358			0.63
Wild type		374 (67%)	Ref.			240 (67%)	Ref.	
Mutated		181 (33%)	1.36 (0.86–2.13)			118 (33%)	1.09 (0.77–1.54	
BRAF	544			0.27	371			0.089
Wild type		478 (88%)	Ref.			315 (85%)	Ref.	
Mutated		66 (12%)	0.63 (0.27–1.45)			56 (15%)	1.43 (0.94–2.17)	
Microsatellite instability	569			0.047	381			0.25
MSI-H		77 (14%)	Ref.			42 (11%)	Ref.	
MSS		492 (86%)	2.43 (0.98–6.01)			339 (89%)	1.39 (0.79–2.46)	
CD8^+^ T-lymphocyte and stroma fraction	598			0.076	394			<0.001
High CD8^+^ fraction and low stroma fraction		282 (47%)	Ref.			138 (35%)	Ref.	
Low CD8^+^ fraction or high stroma fraction		278 (46%*)*	1.41 (0.90–2.23)			222 (56%)	1.36 (0.96–1.94)	
Low CD8^+^ fraction and high stroma fraction		38 (6%)	2.20 (1.05–4.61)			34 (9%*)*	2.86 (1.75–4.69)	
CD8^+^ T-lymphocyte fraction	598			0.25	394			0.028
High (>2.7%)		331 (55%)	Ref.			174 (44%)	Ref.	
Low (<=2.7%*)*		267 (45%)	1.28 (0.84–1.96)			220 (56%)	1.43 (1.04–1.96)	
Stroma fraction	598			0.029	394			0.002
Low (<56%)		511 (85%)	Ref.			324 (82%)	Ref.	
High (>=56%)		87 (15%)	1.75 (1.05–2.92)			70 (18%)	1.74 (1.21–2.50)	

Definition: low-risk stage ΙΙΙ, pathological tumor stage (pT) 3 pathological lymph node stage (pN) 1 with neither lymphatic nor vascular invasion; high-risk stage ΙΙΙ, stage ΙΙΙ pT3 N1 with lymphatic and/or vascular invasion, pT4, or pN2. Abbreviation: HR, hazard risk; CI, confidence interval; Ref, reference; CAP, capecitabine; BEV, bevacizumab; MSI-H, microsatellite instability-high; MSS, microsatellite stable.

### 3.4 High-risk stage ΙΙΙ CRC (pT3N1 L^+^/V^+^, pT4, or pN2)

**Fig. 5:**
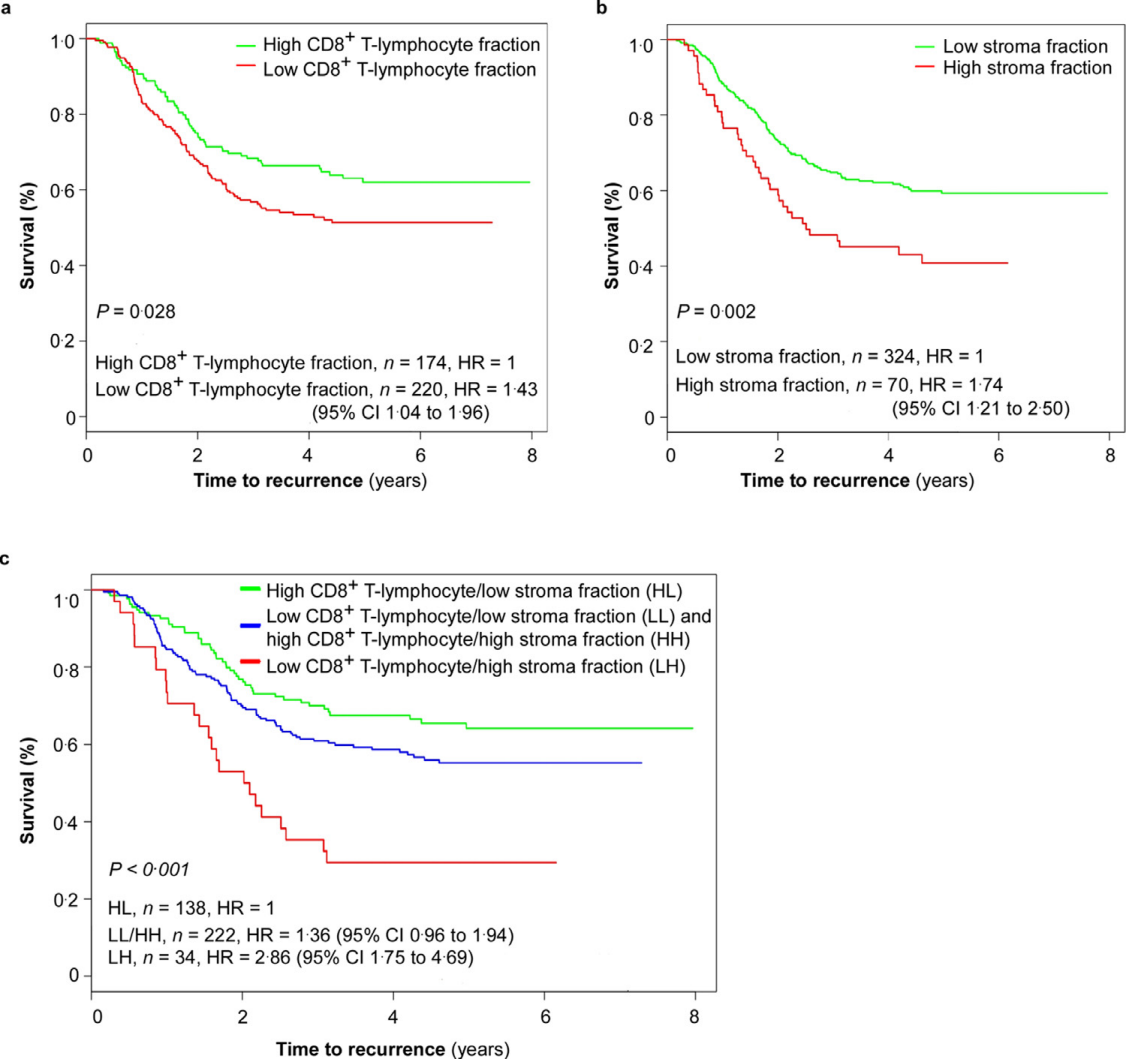
**Kaplan-Meier curves for time to recurrence (TTR) in the high-risk subgroup of the validation set (*n =* 394).** (a) Kaplan-Meier curves for TTR according to CD8^+^ T-lymphocyte fraction in patients with high-risk stage ΙΙΙ colorectal cancer (CRC). 5-year TTR: 62% (95% CI 55–70) for the patients with high CD8^+^ T-lymphocyte fraction versus 51% (95% CI 45–59) for the patients with low CD8^+^ T-lymphocyte fraction (Mantel-Cox log-rank test, *P* = 0.028). (b) Kaplan-Meier curves for TTR according to stroma fraction. 5-year TTR: 59% (95% CI 54–65) for the patients with low stroma fraction versus 41% (95% CI 30–55) for the patients with high stroma fraction (Mantel-Cox log-rank test, *P* = 0.002). (c) Kaplan-Meier curves for TTR according to combined CD8^+^ T-lymphocyte fraction/stroma fraction. 5-year TTR: 64% (95% CI 56–73) for the patients with high CD8^+^ T-lymphocyte fraction/low stroma fraction, 55% (95% CI 49–63) for the patients with combined low CD8^+^ T-lymphocyte fraction/low stroma fraction or combined high CD8^+^ T-lymphocyte fraction/high stroma fraction, and 29% (95% CI 17–50) for the patients with low CD8^+^ T-lymphocyte fraction/ high stroma fraction (Mantel-Cox log-rank test, *P* < 0.001). High-risk stage ΙΙΙ CRC: pathological tumor stage (pT) 3 pathological lymph node stage (pN) 1 with either lymphovascular invasion (L^+^) or venous vascular invasion (V^+^); pT4; or pN2. HR, hazard risk; CI, confidence interval.

**Fig. 6:**
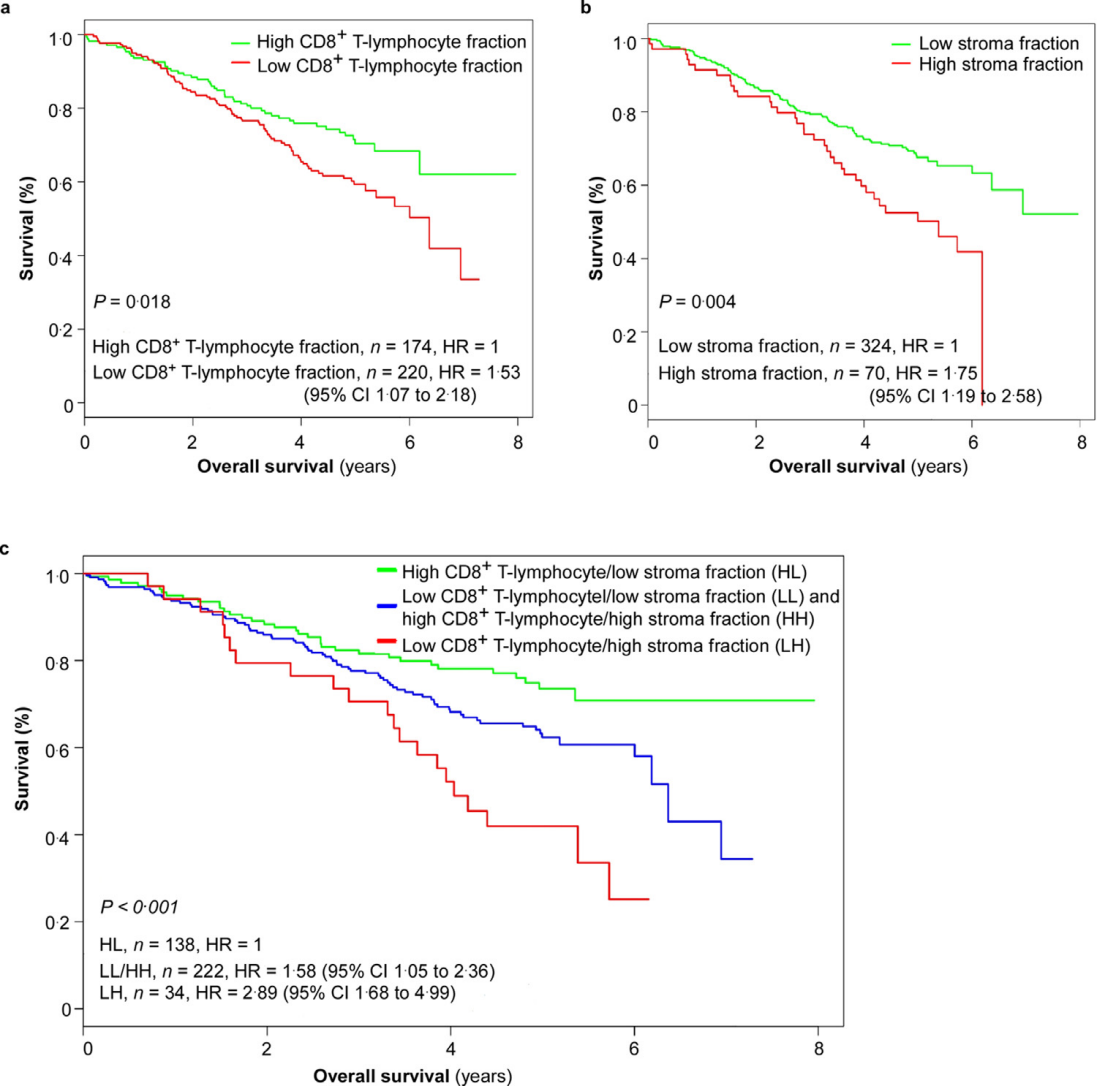
**Kaplan-Meier curves for overall survival (OS) in the high-risk subgroup of the validation set (*n =* 394).** (a) Kaplan-Meier curves for OS according to the CD8^+^ T-lymphocyte fraction in patients with high-risk stage ΙΙΙ colorectal cancer (CRC). 5-year OS: 70% (95% CI 63–78) for the patients with high CD8^+^ T-lymphocyte fraction versus 59% (95% CI 53–67) for the patients with low CD8^+^ T-lymphocyte fraction (Mantel-Cox log-rank test, *P* = 0.018). (b) Kaplan-Meier curves for OS according to stroma fraction. 5-year OS: 69% (95% CI 63–76) for the patients with low stroma fraction versus 56% (95% CI 47–65) for the patients with high stroma fraction (Mantel-Cox log-rank test, *P* = 0.004). (c) Kaplan-Meier curves for OS according to combined CD8^+^ T-lymphocyte fraction/stroma fraction. 5-year OS: 74% (95% CI 66–82) for the patients with high CD8^+^ T-lymphocyte fraction/low stroma fraction, 62% (95% CI 56-70) for the patients with combined low CD8^+^ T-lymphocyte fraction/low stroma fraction or combined high CD8^+^ T-lymphocyte fraction/high stroma fraction, and 42% (95% CI 28–63) for the patients with low CD8^+^ T-lymphocyte fraction/high stroma fraction (Mantel-Cox log-rank test, *P* < 0.001). High-risk stage ΙΙΙ CRC, pathological tumor stage (pT) 3 pathological lymph node stage (pN) 1 with either lymphovascular invasion (L^+^) or venous vascular invasion (V^+^); pT4; or pN2. HR, hazard risk; CI, confidence interval.

For high-risk patients (*n =* 394; stage ΙΙΙ pT3N1 L^+^/V^+^, pT4, or pN2) the combined CD8^+^/stroma marker showed greater predictivity than either marker employed alone. Three discrete prognostic subgroups were defined using this combined marker. 5-year TTR (Fig. 5, Table 3): 64% (95% CI 56–73) for the patients with high CD8^+^ and low stroma (*n =* 138), 55% (95% CI 49–63) for the patients with low CD8^+^ and low stroma or high CD8^+^ and high stroma (*n =* 222), and 29% (95% CI 17–50) for the patients with low CD8^+^ and high stroma (*n =* 34; HR = 2.86, 95% CI 1.75–4.69; Mantel-Cox log-rank test, *P* < 0.001). 5-year OS (Fig. 6, Supplementary Table 1): 74% (95% CI 66–82) for the patients with high CD8^+^ and low stroma, 62% (95% CI 56–70) for the patients with low CD8^+^ and low stroma or high CD8^+^ and high stroma, 42% (95% CI 28–63) for the patients with low CD8^+^ and high stroma (HR = 2.89, 95%CI 1.68–4.99; Mantel-Cox log-rank test, *P* < 0.001).

## 4 Discussion

Analysis of 1041 early stage CRC from a mature clinical trial, showed that the combination of CD8^+^ T-lymphocyte fraction and stroma fraction can improve current prognostic-risk stratification methods, demonstrating the biological importance of the tumor microenvironment in determining outcome. Our results suggest that integrating these two automated objective markers, CD8^+^ T-lymphocyte fraction and stroma fraction, with widely accepted clinicopathological high-risk factors, can help to refine prognostic information for stage Ⅱ/ΙΙΙ CRC. Methodologically, both parameters can be measured automatically using novel quantitative digital microscopy software, adding elements of objectivity, consistency and rapidity to the pathological report.

Our combined prognostic marker, integrating conventional histopathological variables with digital estimates of lymphocyte and stroma fractions, showed excellent prognostic discrimination, stratifying patients with stage Ⅱ/ΙΙΙ CRC into different outcome subgroups. It can identify a good prognostic subgroup with 5-year TTR of 86% (95% CI 83–89). Meanwhile, it also can identify a very poor prognostic subgroup stage ΙΙΙ CRC, with 5-year TTR of only 29% (95% CI 17–50) and a 5-year OS of 42% (95% CI 28–63), despite receiving capecitabine. Furthermore, this integrated prognostic marker can distinguish a relatively good prognostic subgroup from the currently considered high-risk (T4 or N2) stage ΙΙΙ CRC [18]. The combination of high CD8^+^ T-lymphocyte fraction and low stroma fraction can identify a subgroup with a 5-year TTR rate was 64% (95% CI 56–73) from this conventionally accepted high-risk stage ΙΙΙ CRC, even although the patients in the QUASAR 2 study received only single-agent capecitabine.

Although most weight in personalized cancer medicine is given to predictive markers, matching specific anticancer drugs to patients whose tumors express particular chemosensitivity biomarkers, there is a growing role for the application of improved prognostic markers to stratify patients, broadly, into those with a very low chance of recurrence who, in the adjuvant setting, would not benefit from postoperative chemotherapy, and those patients with a substantially higher risk of recurrence who will have a higher absolute benefit from additional chemotherapy. Ideally, prospective clinical trials in which patients were separated into different prognostic groups and then randomized into studies exploring chemotherapy regimes of differing intensity and complexity will be undertaken.

Current evidence suggests a correlation between both CD8^+^ T-lymphocyte and tumoral stroma estimation with therapeutic efficiency of adjuvant chemotherapy. Transcriptional subtyping and CD8 IHC can identify patients with stage Ⅱ/ΙΙΙ CRC more likely to benefit from adjuvant fluorouracil-based chemotherapy [19]. Patients with stage ΙΙΙ CRC of stromal subtype (>50% tumor stromal percentage) trended towards improved disease-free survival (DFS) when receiving CAPEOX (capecitabine and oxaliplatin), whereas those with the immune subtype appeared to benefit more from FOLFOX (fluorouracil, leucovorin, and oxaliplatin) regime [20].

Although we and many others have identified MSI status as a contributory prognostic factor in early stage CRC, it was not sufficiently strong in this dataset to segregate stage II and III disease, and did not add value to the other prognostic markers defined in the multivariate model [17,21]. All patients in this trial received 6 months treatment with capecitabine and this may have confounded the prognostic impact of MSI.

The strengths of the present study included the automatic identification of CD8^+^ T-lymphocyte fraction and stroma fraction using software tools to analyze digital pathology images, which increased the objectivity and repeatability of the analysis process, whilst outperforming conventional genetic biomarkers (KRAS, BRAF). Moreover, this new prognostic marker consists of two morphologic features which are easily extracted from the routine H&E and IHC stained slides, making it quickly estimated and tissue-saving method, compared to current genetic testing.

This study has some limitations. CD8^+^ T-lymphocyte fraction was analyzed using TMA rather than whole sections, focused on intratumoral infiltration rather than the combination of the tumor center and invasive margin. The spatial distribution of T-lymphocytes has been shown to be an important prognostic indicator in some cancers, including CRC, particularly in the Immunocore assay based on quantification of CD3^+^ and CD8^+^ T-lymhocytes densities [6,22,23]. However, a statistically significant correlation between CD3^+^/CD8^+^ T-lymphocyte and CD8^+^ T-lymphocyte fraction in the tumor center and invasive margins was obtained, by measuring lymphocyte fractions in TMA cores and whole section slides in an earlier study [11]. Although the two studies and their patient populations are not directly comparable, it would appear that the integrated biomarker gives a wider dynamic prognostic range (86–29% 5-year TTR) than Immunocore when applied singly (75–57% 5-year DFS for pooled stage Ⅰ-III colon cancer) [6]. In addition, we hypothesize that this integrated biomarker, given its association with accepted, widely reported histopathological variables would increase the likelihood of it being incorporated into standard pathology reports describing early stage CRC.

In summary, measurement of intratumoral CD8^+^ T-lymphocyte and stroma fractions combined with conventional markers of prognosis (*T* and *N* stage, lymphatic and vascular invasion, identified on multivariate modeling) provides superior patient stratification for recurrence and survival for early stage CRC patients.

## Contributors

Conceptualization and supervision: HD, LY and DK. Methodology: HD, DK, TH and DJ. Data collection: MG, DC, DK and TH. Data analysis: TH and DJ. Interpretation: DK and DJ. Writing-original draft: DJ. Writing- review & editing: DK. Verification of the data: HD, LY, DK, TH and DJ. All authors read and approved the final version of the manuscript.

## Data sharing statement

Datasets generated during and analysed during the current study are available from the corresponding authors upon reasonable request.

## Declaration of Competing Interest

DK reports personal fees from Oxford Cancer Biomarkers (Oxford, UK), outside the submitted work. All other authors declare no competing interests.

## Appendix Supplementary materials

Image, application 1

Supplementary Table 1 Univariate analyses of 5-year overall survival (OS) according to clinicopathological features in the validation set (*n =* 1041). Image, application 1
