# Supplementary material for: Automated assessment of CD8+ T-lymphocytes and stroma fractions complement conventional staging of colorectal cancer
Source: eBioMedicine. 2021 Aug 31;71:103547. doi: 10.1016/j.ebiom.2021.103547 (PMC8411014; doi:10.1016/j.ebiom.2021.103547)
Supplement: Supplementary file 1 [file mmc1.pdf]

Supplementary Table 1

Univariate analyses of 5-year overall survival (OS) according to clinicopathological features in the validation set (n=1041).

	Stage II and low-risk stage III				High-risk stage III			
**Variable**	**Total number**	**n (%)**	**HR (95% CI)**	***P*-value**	**Total number**	**n (%)**	**HR (95% CI)**	***P*-value**
Age	598	598 (100%)	1·03 (1·00-1·06)	0·034	394	394 (100%)	1·01 (0·99-1·02)	0·52
Gender	598			0·19	394			0.72
Male		342 (57%)	Ref.			234 (59%)	Ref.	
Female		256 (43%)	0·71 (0·43-1·19)			160 (41%)	1·07 (0·76-1·50)	
Randomised adjuvant treatment	598			0·36	394			0·12
CAP		295 (49%)	Ref.			197 (50%)	Ref.	
CAP+BEV		303 (51%)	1·26 (0·77-2·05)			197 (50%)	1·31 (0·93-1·84)	
Sidedness	578			0·90	387			0·001
Right		321 (56%)	Ref.			226 (58%)	Ref.	
Left		257 (44%)	0·97 (0·58-1·60)			161 (42%)	1·75 (1·24-2·47)	
Number of lymph nodes removed	583			0·013	393			0·60
>=12		453 (78%)	Ref.			307 (78%)	Ref.	
<12		130 (22%)	1·92 (1·14-3·23)			86 (22%)	0·89 (0·59-1·35)	
Histological grade	572			0·48	383			0·17
Well		25 (4%)	Ref.			13 (3%)	Ref.	
Moderate		461 (81%)	2·55 (0·35-18·49)			302 (79%)	1·09 (0·40-2·97)	
Poor		86 (15%)	3·18 (0·41-24·62)			68 (18%)	1·62 (0·57-4·63)	
KRAS	555			0·56	358			0·41
Wild type		374 (67%)	Ref.			240 (67%)	Ref.	
Mutated		181 (33%)	1·17 (0·69-1·97)			118 (33%)	1·17 (0·81-1·69)	
BRAF	544			0·031	371			0·093
Wild type		478 (88%)	Ref.			315 (85%)	Ref.	

Mutated		66 (12%)	1·98 (1·05-3·74)			56 (15%)	1·47 (0·93-2·32)	
Microsatellite instability	569			0·50	381			0·74
MSI-H		77 (14%)	Ref.			42 (11%)	Ref.	
MSS		492 (86%)	0·79 (0·40-1·56)			339 (89%)	1·11 (0·61-2·01)	
$CD8^{+}$ T-lymphocyte and stroma fraction	598			0·43	394			<0·001
High CD8 fraction and low stroma fraction		282 (47%)	Ref.			138 (35%)	Ref.	
Low CD8 fraction or high stroma fraction		278 (46%)	1·39 (0·83-2·32)			222 (56%)	1·58 (1·05-2·36)	
Low CD8 fraction and high stroma fraction		38 (6%)	1·41 (0·54-3·70)			34 (9%)	2·89 (1·68-4·99)	
$CD8^{+}$ T-lymphocyte fraction	598			0·70	394			0·018
High (>2.7%)		331 (55%)	Ref.			174 (44%)	Ref.	
Low (<=2.7%)		267 (45%)	1·10 (0·68-1·79)			220 (56%)	1·53 (1·07-2·18)	
Stroma fraction	598			0·12	394			0·004
Low (<56%)		511 (85%)	Ref.			324 (82%)	Ref.	
High (>=56%)		87 (15%)	1·59 (0·88-2·88)			70 (18%)	1·75 (1·19-2·58)	

Definition: low-risk stage III, pathological tumour stage (pT) 3 pathological lymph node stage (pN) 1 with neither lymphatic nor vascular invasion; high-risk stage III, stage III pT3 N1 with lymphatic and/or vascular invasion, pT4, or pN2. Abbreviation: HR, hazard risk; CI, confidence interval; Ref, reference; CAP, capecitabine; BEV, bevacizumab; MSI-H, microsatellite instability-high; MSS, microsatellite stable.
