# Supplementary material for: Automated assessment of CD8+ T-lymphocytes and stroma fractions complement conventional staging of colorectal cancer
Source: eBioMedicine. 2021 Aug 31;71:103547. doi: 10.1016/j.ebiom.2021.103547 (PMC8411014; doi:10.1016/j.ebiom.2021.103547)
Supplement: Supplementary file 2 — Supplementary Table 1 Univariate analyses of 5-year overall survival (OS) according to clinicopathological features in the validation set (n = 1041). [file mmc2.docx]

**Caption for supplementary material**

**Automated assessment of CD8^+^ T-lymphocyte and stroma fractions complement conventional staging of colorectal cancer**

Dan Jiang^1,2,3^, Tarjei S. Hveem^4^, Mark Glaire^5^, David N. Church^5,6^, Håvard E. Danielsen^3,4,7^ †, Li Yang^1,8^† and David J. Kerr^1,3^

**Authors' Affiliations:**

1. Sichuan University-University of Oxford Huaxi Joint Centre for Gastrointestinal Cancer, West China Hospital, Sichuan University, Chengdu, China.
2. Department of Pathology, West China Hospital, Sichuan University, Chengdu, China
3. Radcliffe Department of Medicine, University of Oxford, Oxford, United Kingdom.
4. Institute for Cancer Genetics and Informatics, Oslo University Hospital, Oslo, Norway
5. Wellcome Centre for Human Genetics, University of Oxford, Oxford, United Kingdom
6. NIHR Oxford Biomedical Research Centre, Oxford University Hospitals NHS Foundation Trust, John Radcliffe Hospital, Oxford, United Kingdom
7. Department of Informatics, University of Oslo, Oslo, Norway
8. Department of Gastroenterology and Hepatology, West China Hospital, Sichuan University, Chengdu, Sichuan, China

†Authors contributed equally as co-corresponding authors.

**Supplementary Table 1 Univariate analyses of 5-year overall survival (OS) according to clinicopathological features in the validation set (n=1041).**
